# Poultry Farm Vulnerability and Risk of Avian Influenza Re-Emergence in Thailand

**DOI:** 10.3390/ijerph110100934

**Published:** 2014-01-09

**Authors:** Marc Souris, Dubravka Selenic, Supaluk Khaklang, Suwannapa Ninphanomchai, Guy Minet, Jean-Paul Gonzalez, Pattamaporn Kittayapong

**Affiliations:** 1 Center of Excellence for Vectors and Vector Borne Diseases, Faculty of Science, Mahidol University at Salaya, Nakhon Pathom 73170, Thailand; E-Mails: dselenic@hotmail.com (D.S.); s_khaklang@hotmail.fr (S.K.); ninphanomchai_2@hotmail.com (S.N.); g.minet@yahoo.fr (G.M.); jpgonzalez@metabiota.com (J.-P.G.); pkittayapong@msn.com (P.K.); 2 UMR_D 190 “Emergence des Pathologies Virales”, IRD French Institute of Research for Development, Aix-Marseille University, EHESP French School of Public Health, Marseille 13385, France; 3 Asian Institute of Technology, P.O. Box 4, Klong Luang, Pathumthani 12120, Thailand; 4 Metabiota, Washington, DC 20009, USA

**Keywords:** HPAI H5N1, vulnerability assessment, poultry production system, Thailand

## Abstract

Highly pathogenic avian influenza (HPAI) remains of concern as a major potential global threat. This article evaluates and discusses the level of vulnerability of medium and small-scale commercial poultry production systems in Thailand related to avian influenza virus re-emergence. We developed a survey on 173 farms in Nakhon Pathom province to identify the global level of vulnerability of farms, and to determine which type of farms appears to be more vulnerable. We used official regulations (the Good Agricultural Practices and Livestock Farm Standards regulations) as a reference to check whether these regulations are respected. The results show that numerous vulnerability factors subsist and could represent, in case of HPAI re-emergence, a significant risk for a large spread of the disease. Bio-security, farm management and agro-commercial practices are particularly significant on that matter: results show that these practices still need a thorough improvement on a majority of farms. Farms producing eggs (especially duck eggs) are more vulnerable than farms producing meat. Those results are consistent with the type of farms that were mostly affected during the 2004–2008 outbreaks in Thailand.

## 1. Introduction

The first outbreaks of highly pathogenic avian influenza caused by viruses of the H5N1 subtype (H5N1 HPAI) were detected in geese in 1996 in the Chinese province of Guangdong and in 1997 in Hong Kong. Until today, 54 countries in Asia, Europe and Africa have reported H5N1 HPAI outbreaks among poultry; outbreaks in poultry farms have been reported in every country in South East Asia (SEA), except Brunei, Singapore and the Philippines. A number of preventive measures have been implemented to keep the disease under control; however, it reappears sporadically and is now enzootic in some countries [1]. H5N1 HPAI remains of concern as a major potential global threat.

Thailand officially declared it first H5N1 HPAI outbreak in a layer farm in the central region on January 2004. From 2004 until 2008, H5N1 HPAI was laboratory confirmed in 1,890 poultry flocks in Thailand, and 17 human deaths out of 25 cases were reported [2]. More than 63 million birds had been culled due to control measures. The epidemics had been determined as a major threat to the poultry industry and caused reduction in chicken production and consumption with a huge economic impact [3]. The last occurrence of H5N1 HPAI outbreak in Thailand was declared to the World Organization for Animal Health (OIE) in November 2008, and, since then no laboratory-confirmed outbreaks have been reported. Nevertheless, studies in ducks revealed serological evidence of H5 infection in 2010 [4], and in some neighboring countries re-emergence of H5N1 HPAI is still ongoing.

Thailand’s poultry industry has grown rapidly over the twenty years leading up to 2004, especially in the broiler production area. In 2002, the country was the fourth largest exporter of poultry meat [5]. The poultry production system in Thailand can be classified according to Food and Agriculture Organization (FAO) categorization as: sector 1, large-scale industrial production, with high bio-security; sector 2, semi-industrial production (commercial poultry production system with moderate to high bio-security and birds/products usually marketed commercially); sector 3, small to medium scale semi-commercial poultry production (with low to minimal bio-security and birds/products usually marketed at local bird markets) and; sector 4, smallholder backyard farming with minimal or no bio-security [6].

Vulnerability factors associated with HPAI outbreaks have been the subject of many investigations. Agro-commercial activities, human behaviors, transports and poultry smuggling have been identified as major factors of local dispersion of the virus in Thailand [2,7,8,9,10,11]. To certificate safety poultry production and to minimize poultry farms vulnerability, the Bureau of Livestock Standard and Certification of Department of Livestock Development (DLD, Ministry of Agriculture, Bangkok, Thailand) set standards for Good Agricultural Practice (GAP) and Livestock Farm Standards (LFS), with regular surveillance and yearly recertification audits of poultry farms. Variables based on GAP and LFS comprise eight main sections (for broiler and meat ducks farms) or nine (for layer farms): general condition of the farm, poultry feed, water quality, farm management, animal health, poultry welfare management, environmental management, data recording and productivity management for layers farms. Immediately after the first wave of HPAI outbreaks, in 2004, DLD was immensely concerned in increasing farms bio-security and preparing new policy measures [12]. For example, farmers were required to improve open poultry housing to a closed evaporative system. However these measures were not fully implemented, and still, in certified poultry farm standards, both (open and closed-evap) houses are allowed [13]. No study evaluating the actual level of vulnerability of poultry farms in Thailand using official GAP and LFS regulations was published recently. A study was performed in 2011 in three provinces, but focused on the main pathway of entry of influenza viruses onto farms with different production methods [14].

This article describes and discusses the actual level of vulnerability of medium and small-scale poultry commercial production system (sector 2 and 3) related to HPAI risk in Thailand. We use official GAP and LFS regulations as the references to identify the overall level of vulnerability of poultry farms, checking on farms whether these regulations are respected. The aim of this article is to evaluate the global level of vulnerability, to highlight the most common factors of vulnerability, and to determine which type of farms appears to be more vulnerable. Also, spatial analysis is performed in order to find any geographical pattern of vulnerability susceptible to increase the risk of HPAI farm-to-farm transmission.

## 2. Material and Methods

### 2.1. Field Survey in Nakhon Pathom Province

**Figure 1:**
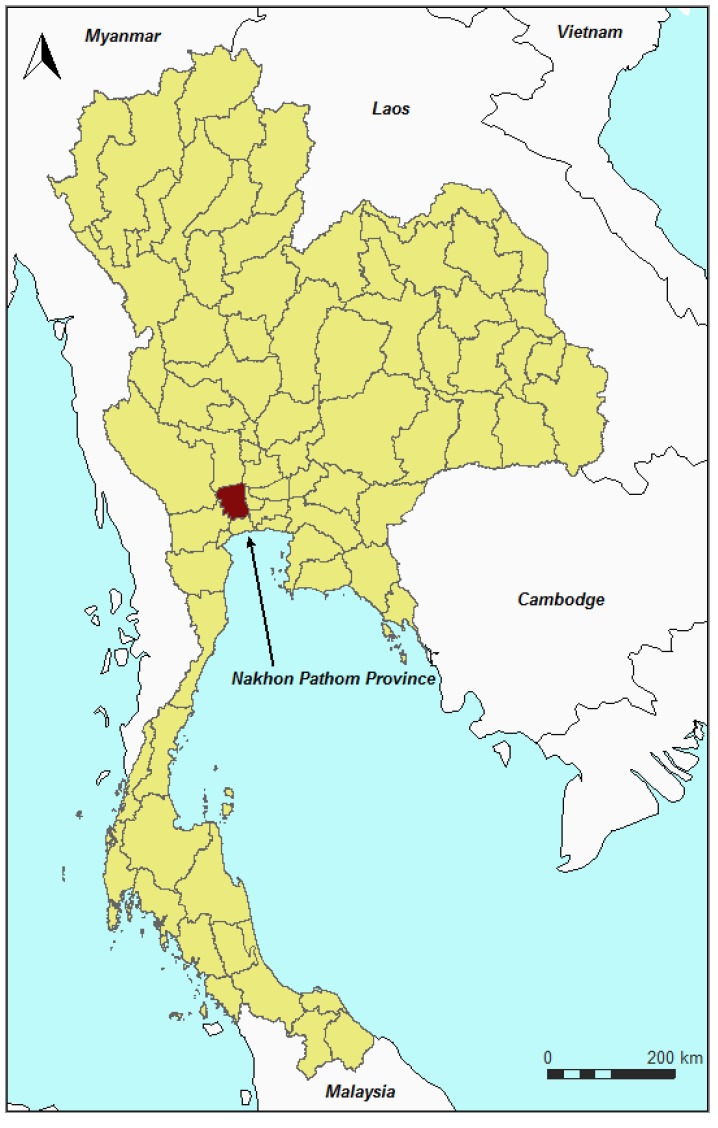
Nakhon Pathom province in Thailand.

We conducted a field survey supported by a comprehensive semi-structured questionnaire in the 173 registered commercial poultry farms in the Nakhon Pathom province, Thailand (Figure 1) from March to September 2011. The Nakhon Pathom province, in the central region of Thailand, has been selected for our study because of its extensive agriculture activities and for logistic reasons. The province (2,168 km²) consists of seven districts, 106 sub-districts and 870 villages, with nearly 1,000,000 inhabitants in 2011. Nakhon Pathom is nationally ranked 7th and 2nd for its broiler and duck meat production, 6th and 8th for chicken and duck layer farm (DLD, 2008). It has 212 registered poultry farms belonging to sector 1 to 3 (DLD, 2010). Farms belonging to sector 1 were not included in the study as they are considered to have limited vulnerability because they implement high level operating procedures to reduce the risk of viral incursions [6]. The sector 4 farms (backyard production with minimal bio-security and birds/products consumed locally) were also not included in this study as we considered that outdoor backyard production with minimal or no bio-security measures cannot be equally compared to farms [9,15].

The present survey included all 173 registered commercial poultry farms of the province belonging to sector 2 and sector 3 in 2011: 96 broiler farms, 33 farms of laying hens, 28 ducks meat farms and, 16 farms of laying ducks. The location of each farm entrance was recorded by GPS. An environmental description was conducted from the farm entrance. A questionnaire was used to describe agricultural practices from owners or workers; all interviews were conducted in Thai language. Questionnaires were addressed directly, without an appointment, to owners or workers, at the entrance of the farms.

### 2.2. HPAI in the Nakhon Pathom Province, 2004–2008

**Figure 2:**
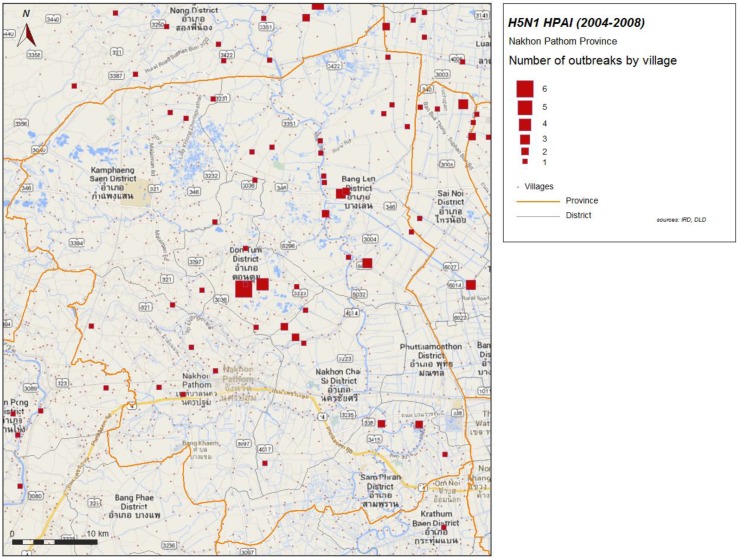
H5N1 HPAI outbreaks by village, 2004–2008 in Nakhon Pathom province.

**Figure 3:**
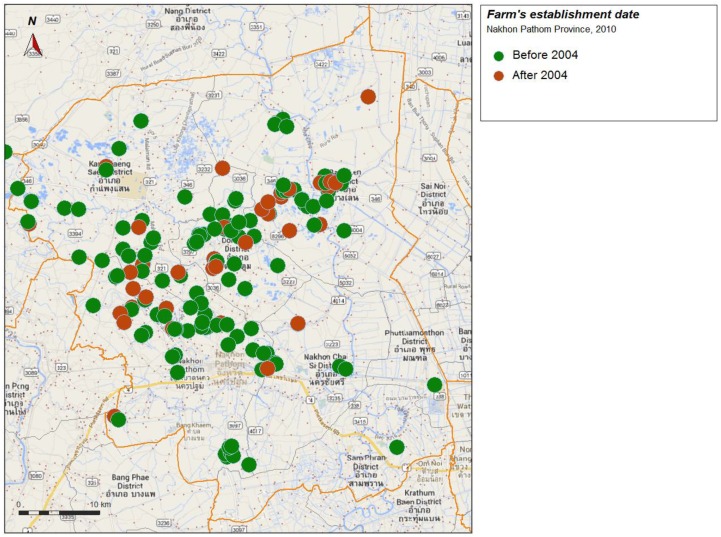
Farms in the survey and date of establishment (green: before 2004, brown: after 2004).

Forty one H5N1 HPAI laboratory confirmed outbreaks were reported in Nakhon Pathom from 2004 to 2005 (Figure 2). The province has suffered strong economic effects, like the neighboring province of Suphanburi where H5N1 HPAI emerged in late 2003 [2]. Of the 173 farms from sector 2 and 3 of Nakhon Pathom inspected in 2011, 43 were established after 2004 (Figure 3).

### 2.3. Vulnerability Factors, Vulnerability Scores, Statistical and Spatial Analysis

Vulnerability factors in the present study are limited to factors described in DLD, GAP and LFS regulations and are listed in Table 1. All factors are Boolean (yes/no answers). They were grouped into five categories, as per DLD regulations:

Cat. 1: general condition of the farm, environmental and geographical description, environmental and farm condition, animal health and poultry welfare management;
Cat. 2: agricultural and agro-commercial practice, slaughtering and selling bird on the farm, to who birds are sold, who organize transports, frequency of transport, “all-in-all-out” practice, restocking time;
Cat. 3: bio-security measures and farm management, including transports, restocking practice, manure utilization, water management, rodent and insect control, frequency of disinfection, workers routine and hygiene practice, contact between farms;
Cat. 4: agro-commercial practice of layers farms: egg transportation, disinfection of egg trays, percentages of sale direct to contractors, sale through aggregators;
Cat. 5: knowledge of the local farmer’s regarding HPAI and verification of their commitment to HPAI risk management;

We assigned a weight to each vulnerability factor in order to calculate global vulnerability scores as additive model of vulnerability. Scores are then unique values affected to each farm and make vulnerability evaluation more synthetic.

**Table 1:** Collected factors of vulnerability and related category and weight.

n°	Factors of Vulnerability	Category *	Weight **
1	Distance to main road less than 50 m	1	7
2	No fence at all around the farm	1	9
3	Easy entry to the farm	1	8
4	A pond is on the farm or next to the farm	1	6
5	Open air house on fish pond	1	6
6	Dead fowl used as animal food	2	6
7	Sale of dead birds	2	9
8	Carcass of dead birds throw away	2	7
9	Domestic cats and dogs living on the farm	1	4
10	Stray dogs/cats on farm	1	8
11	Backyard poultry on the farm	1	9
12	Presence of other birds	1	5
13	Fighting cocks on the farm	1	4
14	Presence of exotic birds in cage	1	2
15	Presence of other farm animals (pigs, cow, horse, rabbit, boar)	2	4
16	Frog breeding on the farm	2	2
17	No rodent control	3	6
18	No use of insecticides	3	4
19	Outsider are permitted to enter the farm	3	9
20	Frequent entry/exit from farm more than two times per day	3	9
21	Workers do not have working clothing	3	9
22	Same employees work on several units on the same farm	3	5
23	No farm disinfection	3	9
24	Disinfectant barrier at farm entrance does not exist	3	9
25	Footbath next to poultry house does not exist	3	8
26	Vehicles are not disinfected before entering	3	9
27	Dealers/Drivers free movement without changing clothes	3	9
28	Poultry houses are not disinfected at end of production	3	9
29	Different age groups in same building (no all in/all out)	2	9
30	Pond water used for animal drinking	1	7
31	Manure not properly stored	3	5
32	Manure used as animal feed	2	3
33	Use of wet and fresh litter	2	7
34	Not daily cleaning of rearing equipment	3	4
35	Cleaning of equipment only at the end of production cycle	3	6
36	Food delivered by different suppliers	2	7
37	Easy access to food storage for rodents and wild birds	1	8
38	Poultry sold to trader	2	9
39	Restocking before 14 days gap	3	9
40	Slaughtering on the farm without proper hygiene facilities	3	3
41	Egg trays not disinfected at the farm	4	6
42	Eggs are sold on the farm to villagers	4	7
43	Eggs sold to different dealers	4	9
44	Farmers are not informed about AI	5	9
45	Farmers are not concerned about AI as dangerous disease	5	9

Notes: * Categories: 1: Farm General Conditions; 2: Agricultural & agro-commercial practices; 3: Bio-security measures and farm management; 4: Layers farms Agro-commercial practices; 5: AI farmer knowledge. ** Weight assigned to each factor to calculate farm vulnerability scores by linear combination.

A vulnerability score for a farm is calculated as a linear weighted sum, by adding the weight of a factor when this factor is positive (yes answer) for the farm. The scores are scaled (divided by the sum of weights) to fit between 0 (lowest) and 1 (highest):

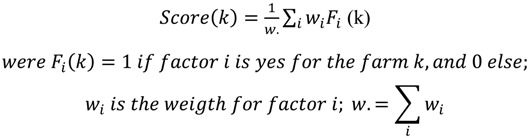



(k) The weights correspond to our appreciation of the relative level of importance between factors in term of vulnerability, as experts in this field. As always when using scores based on weights, we assume that our appreciation is subjective and that significant variation in the weighting may occurs if given by different experts.

For each farm, several scores were calculated:

- A score for each category (management and environmental conditions, agricultural practices, bio-security measures, security measures related to egg production, knowledge of the farmer);
- An overall score of vulnerability considering all variables but eliminating variables related to egg production in layer and meat farms;
- An overall score of vulnerability considering all variables including variables related to egg production. Although variables associated with the eggs production are vulnerability factors for the farms producing eggs and are not relevant for farms producing birds, they were included in the calculation of the overall indicator of vulnerability: egg production increases the potential vulnerability with more potentially “at risk” practices and behaviors.

Statistical results by factor were calculated (proportion of positive answers among farms and standard deviation). Multivariate analysis was performed to find relationships between factors (correlations analysis, principal component analysis). Exposure analysis was also performed to find significative differences of vulnerability related to poultry type (duck or chicken) or poultry production (meat or egg) (null hypothesis: no vulnerability difference can be detected between groups; alternative hypothesis: a difference can be detected between groups). We class the scores in two categories, not vulnerable (Score < 0.5) and vulnerable (Score ≥ 0.5) to perform exposure analysis, considering the type (poultry type, production type) as risk factor. We calculated *p*-values for Student *t*-test, odds-ratios and adjusted odds-ratios (confidence intervals calculated at 5% type I error), and *p*-values for Chi-square, Mantel-Haenszel Chi-square and Fisher-Snedecor tests [16].

Spatial analysis of scores was also performed in order to highlight any relationship of vulnerability with environmental condition or geographic organization of space, and to find any spatial pattern of vulnerability susceptible to increase the risk of HPAI farm-to-farm transmission. We performed global spatial autocorrelation using Moran’s I index (relationship between value and distance between farms), local spatial association analysis using Getis-Ord LISA index (relationship between the value of a farm and the value of neighbor farms) and search of spatial clusters using spatial scan method [17,18,19]. Data were mapped, statistically and spatially analyzed with the SavGIS geographic information system software [20].

### 2.4. Ethics Statement

In this study, no samples (biological or not) were collected. Farm environmental description and interviews were always conducted anonymously outside the farms. No participant names were recorded, and data were analyzed anonymously. Ethics approval was therefore deemed unnecessary by the Mahidol University IRB.

## 3. Results and Discussion

### 3.1. Results

All factors were recorded for the 173 farms in the survey, without any missing data. The consent to participate in the door-to-door interview was 100%, and no specific problems occur in the process of data collection (a pilot survey was first carried out in 2010 to adjust the questionnaire used to describe agricultural practices).

Statistical results by factors (proportion of yes answers, standard deviation) are given in Table 2. Scores of vulnerability are presented in Table 3. Category 3 (bio-security measures and farm management) is the one with the worst average score.

**Table 2:** Results of the survey by factor: proportion of yes answers, standard deviation, results by type of production (meat or layer) or type of bird (chicken or duck).

n°	Vulnerability factors	All farms	Sdt. Dev.	Meat farms	Layer farms	Chicken farms	Duck farms
1	Distance to main road less than 50 m	0.18	0.38	0.16	0.22	0.18	0.18
2	No fence at all around the farm	0.03	0.18	0.04	0.02	0.05	0.00
3	Easy entry to the farm	0.76	0.43	0.74	0.82	0.73	0.86
4	A pond is on the farm or next to the farm	0.75	0.43	0.68	0.92	0.69	0.93
5	Open air house on fish pond	0.11	0.31	0.12	0.06	0.14	0.02
6	Dead fowl used as animal food	0.53	0.50	0.50	0.57	0.50	0.59
7	Sale of dead birds	0.01	0.11	0.01	0.00	0.02	0.00
8	Carcass of dead birds throw away	0.08	0.27	0.08	0.08	0.09	0.07
9	Domestic cats and dogs living on the farm	0.84	0.37	0.82	0.88	0.89	0.68
10	Stray dogs/cats on farm	0.27	0.45	0.32	0.14	0.26	0.32
11	Backyard poultry on the farm	0.18	0.38	0.15	0.26	0.16	0.25
12	Presence of other birds	1.00	0.00	1.00	1.00	1.00	1.00
13	Fighting cocks on the farm	0.05	0.22	0.06	0.02	0.05	0.07
14	Presence of exotic birds in cage	0.04	0.20	0.05	0.02	0.04	0.05
15	Presence of other farm animals (pigs, cow, horse, rabbit, boar)	0.26	0.44	0.26	0.24	0.29	0.18
16	Frog breeding on the farm	0.05	0.210	0.06	0.02	0.06	0.00
17	No rodent control	0.14	0.35	0.10	0.26	0.12	0.20
18	No use of insecticides	0.94	0.23	0.95	0.89	0.94	0.95
19	Outsider are permitted to enter the farm	0.37	0.48	0.31	0.51	0.34	0.45
20	Frequent entry/exit from farm more than two times per day	0.67	0.47	0.60	0.84	0.66	0.70
21	Workers do not have working clothing	0.93	0.25	0.94	0.90	0.92	0.95
22	Same employees work on several units on the same farm	0.38	0.49	0.48	0.14	0.38	0.39
23	No farm disinfection	0.41	0.49	0.37	0.51	0.38	0.50
24	Disinfectant barrier at farm entrance does not exist	0.94	0.24	0.93	0.96	0.91	1.00
25	Footbath next to poultry house does not exist	0.97	0.17	0.98	0.96	0.96	1.00
26	Vehicles are not disinfected before entering	0.55	0.50	0.50	0.67	0.51	0.66
27	Dealers/Drivers free movement without changing clothes	1.00	0.00	1.00	1.00	1.00	1.00
28	Poultry houses are not disinfected at end of production	0.02	0.13	0.01	0.04	0.01	0.05
29	Different age groups in same building (no all in/all out)	0.02	0.13	0.01	0.04	0.01	0.05
30	Pond water used for animal drinking	0.04	0.20	0.01	0.12	0.00	0.16
31	Manure not properly stored	0.03	0.18	0.02	0.08	0.02	0.07
32	Manure used as animal feed	0.57	0.50	0.56	0.61	0.70	0.23
33	Use of wet and fresh litter	0.29	0.46	0.15	0.67	0.31	0.25
34	Not daily cleaning of rearing equipment	0.27	0.44	0.27	0.26	0.34	0.05
35	Cleaning of equipment only at the end of production cycle	0.27	0.44	0.27	0.26	0.34	0.05
36	Food delivered by different suppliers	0.07	0.25	0.07	0.06	0.07	0.07
37	Easy access to food storage for rodents and wild birds	0.83	0.38	0.86	0.73	0.78	0.95
38	Poultry sold to trader	0.39	0.49	0.35	0.49	0.39	0.39
39	Restocking before 14 days gap	0.22	0.42	0.25	0.16	0.27	0.09
40	Slaughtering on the farm without proper hygiene facilities	0.04	0.20	0.06	0.00	0.05	0.00
41	Egg trays not disinfected at the farm	0.10	0.31	0.00	0.37	0.05	0.27
42	Eggs are sold on the farm to villagers	0.21	0.41	0.00	0.73	0.19	0.27
43	Eggs sold to different dealers	0.15	0.36	0.00	0.51	0.14	0.18
44	Farmers are not informed about AI	0.01	0.13	0.01	0.02	0.01	0.05
45	Farmers are not concerned about AI as dangerous disease	0.63	0.48	0.72	0.41	0.64	0.59

**Table 3:** Summary statistics for five categories of vulnerability scores of commercial poultry farms in Nakhon Pathom, Thailand, 2011.

Vulnerability Score Category	Min.	Max.	Mean	Median	Sdt. Dev.
General characteristics of the farm	0.11	0.66	0.39	0.37	0.11
Agricultural and commercial practices	0	0.54	0.20	0.19	0.12
Biosecurity measures, farm management	0.25	0.79	0.52	0.52	0.10
Layers farms practices	0	1	0.16	0	0.29
AI farmer knowledge	0	0.5	0.32	0.5	0.24
Global score	0.17	0.58	0.38	0.37	0.07

Systematic correlation analysis did not reveal strong relationships between vulnerability factors. A multivariate principal component analysis with all the 45 factors of the survey (Table 1) shows a first component explaining only 10% of the total variance.

Analysis of vulnerability by type of production (meat or layer) revealed that excessive vulnerability correlates with farms producing eggs (Table 4). The type of production is very significant for vulnerability since factors associated with eggs production may strongly contribute to increasing vulnerability. Furthermore, the same results are found whenever we take into account the vulnerability factors directly related to egg production.

**Table 4:** Analysis of vulnerability scores by type of production (meat or layer).

Vulnerability Score Category	Meat *	Layer	*p*-value (T)	OR	Chi^2^
General characteristics of the farm	0.38	0.40	0.17	1.32 [0.52, 3.31]	0.34
Agricultural and commercial practices	0.18	0.27	0.00005	3.77 [1.63, 8.69]	10
Biosecurity measures, farm management	0.51	0.55	0.001	5.64 [2.27, 14.01]	16
AI farmer knowledge	0.37	0.21	0.00006	0.27 [0.14, 0.54]	14
Global score	0.36	0.43	<0.000001	13.24 [4.12, 42.51]	27
Score w/t cat. 4	0.39	0.42	0.0006	5.53 [2.29, 13.32]	16

Notes: * Reference group. T: Student. Chi^2^: Chi square value.

Analysis of vulnerability by poultry type (duck or chicken) shows no significant differences (Table 5). With the overall score classified into two classes (not vulnerable, s < 0.5 and vulnerable, s ≥ 0.5), the odd ratio adjusted by poultry production doesn’t show a significant difference between classes.

**Table 5:** Analysis of vulnerability scores by poultry type (chicken or duck), with OR adjusted by production type (meat or layer), and Mantel-Haenszel Chi^2^.

Vulnerability Score Category	Chicken *	Duck	*p*-value (T)	OR (Ajusted)	Chi^2^ (MH)
General characteristics of the farm	0.37	0.43	0.0028	1.25 [0.46, 3.43]	0.15
Agricultural and commercial practices	0.21	0.18	0.056	1.35 [0.55, 3.32]	0.31
Biosecurity measures, farm management	0.51	0.53	0.19	0.81 [0.27, 2.44]	0.22
Layers farms practices	0.13	0.23	0.006	0.93 [0.28, 3.10]	0.01
AI farmer knowledge	0.32	0.32	0.42	1.09 [0.51, 2.32]	0.05
Global score	0.38	0.40	0.03	1.41 [0.65, 3.06]	0.81
Score w/t cat. 4 (Layers farms practices)	0.39	0.41	0.08	2.28 [0.89, 5.86]	3.06

Notes: * Reference group. T: Student. Chi^2^ (MH): Mantel-Haenszel Chi square value.

Finally, the study of scores using all possible combinations between production type (meat or layer) and poultry type (duck or chicken) shows that farms producing duck eggs are the most vulnerable in all categories, with a difference between groups always significant at a 5% type I error (Table 6).

**Table 6:** Analysis by poultry type and production type.

Vulnerability Score	All	Meat		Layer		*p*-value (F *)
		Chicken	Duck	Chicken	Duck	
General characteristics of the farm	0.39	0.37	0.42	0.38	0.44	0.046
Agricultural and commercial practices	0.20	0.19	0.15	0.28	0.24	0.00003
Biosecurity measures, farm management	0.52	0.51	0.50	0.53	0.58	0.02
Layers farms practices	0.16	0.00	0.00	0.50	0.62	<0.00001
AI farmer knowledge	0.32	0.36	0.37	0.21	0.22	0.002
Global score	0.38	0.36	0.36	0.42	0.46	<0.00001
Score w/t cat.4 (Layers farms practices)	0.40	0.39	0.39	0.41	0.44	0.02

Note: * F: Fisher-Snedecor.

**Figure 4:**
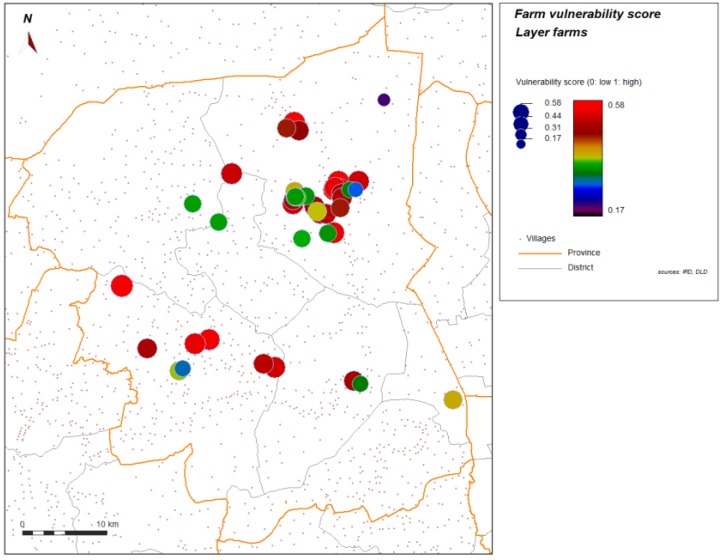
Score of vulnerability, layer farms sector 2 and 3, Nakhon Pathom province, Thailand. Level of vulnerability is given by color graduation and symbol width.

**Figure 5:**
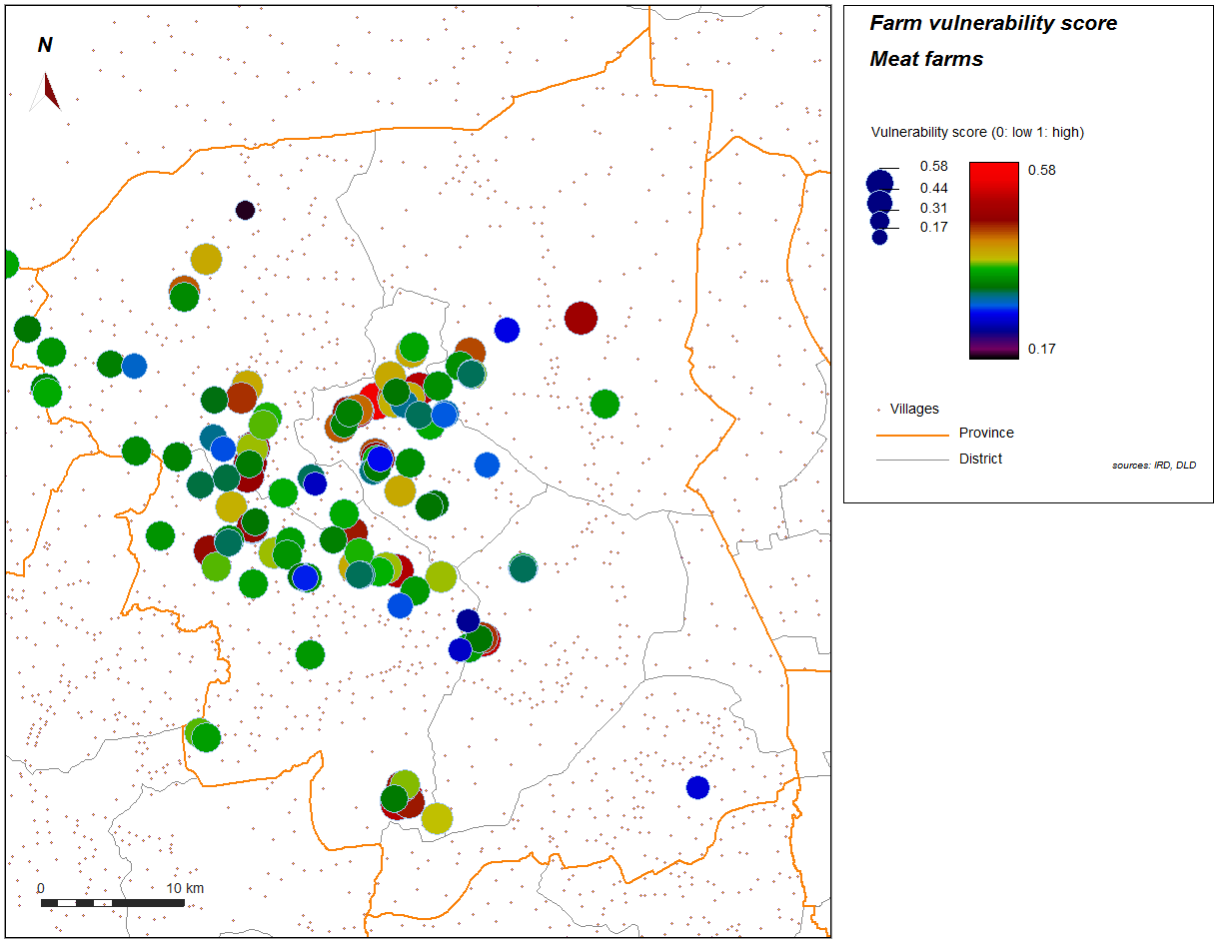
Score of vulnerability, meat farms sector 2 and 3, Nakhon Pathom province, Thailand. Level of vulnerability is given by color graduation and symbol width.

**Figure 6:**
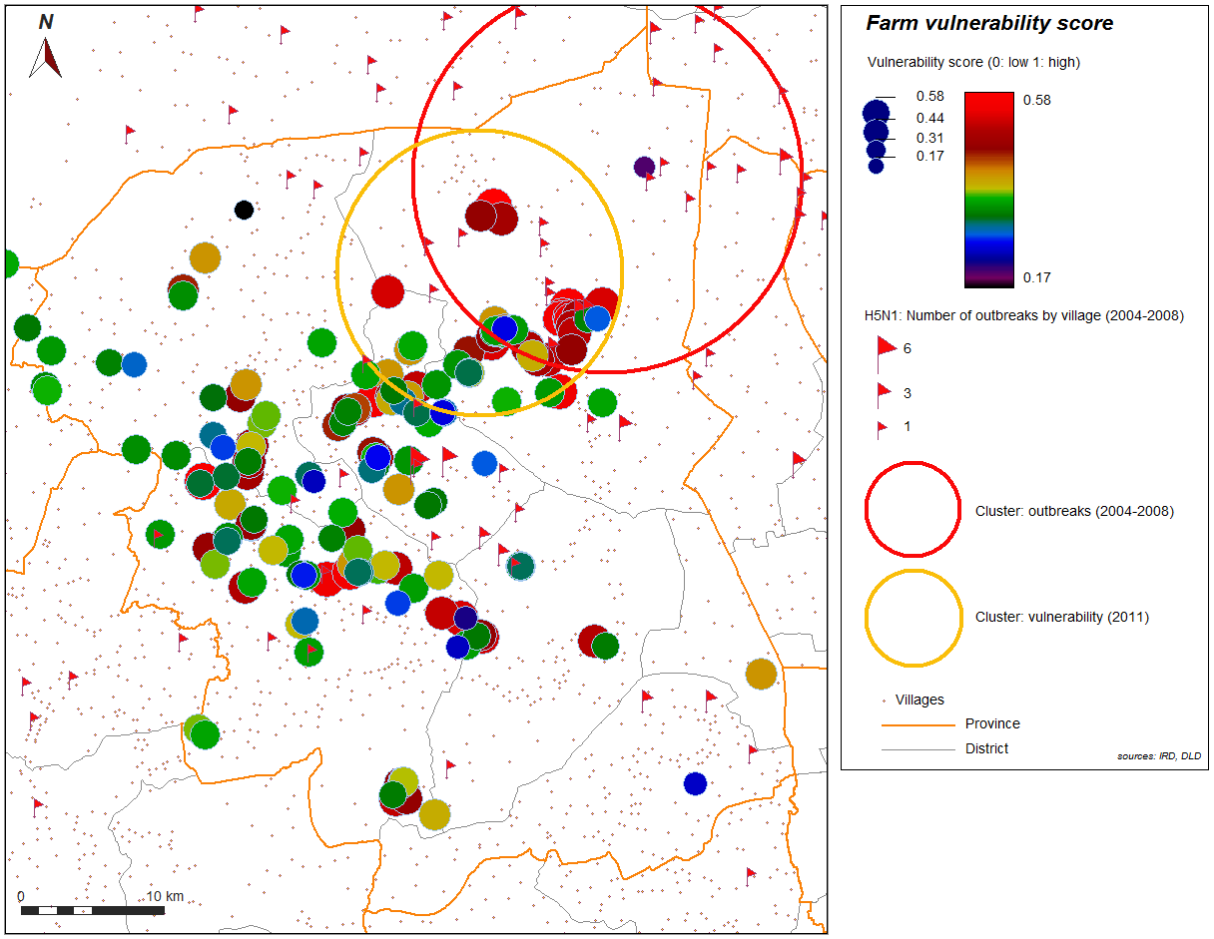
Most significant cluster of vulnerability (yellow), farms sector 2 and 3, and most significant cluster (red) of 2004–2008 H5N1 HPAI outbreaks, Nakhon Pathom province, Thailand.

**Figure 7:**
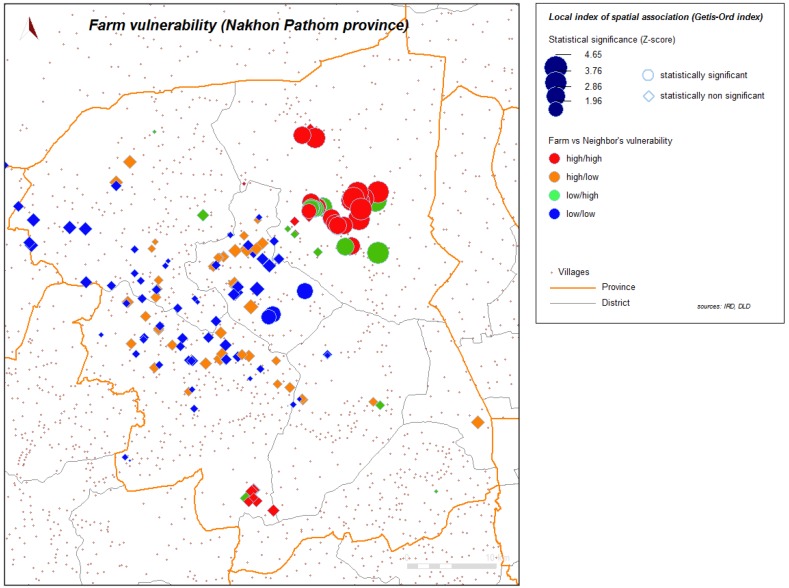
Local index of spatial association (Getis-Ord) for vulnerability, farms sector 2 and 3, Nakhon Pathom province, Thailand.

Spatial analysis was performed to help in identifying further contributing factors in environment or geographic context or organization of space. Figure 4 and Figure 5 show the spatial distribution of the vulnerability score for layer farms and for meat farms. Significant global spatial autocorrelation of global vulnerability score (Z-score > 1.96) is found within a short radius for the spatial weight used in the spatial autocorrelation index (search radius up to 10 km). With automatic cluster detection using a spatial statistic scan, we found a most significant spatial cluster for the vulnerability score in our study, and this cluster is spatially close to the most significant cluster of the H5N1 HPAI cases that occurred during the epidemics from 2004 to 2008, and automatically detected with the same technique (Figure 6). Local spatial association is also found (Figure 7), showing again a clustered pattern, especially for high level of vulnerability.

Spatial analysis performed by separating farms into two groups by poultry production (meat or layer) showed no significant spatial clustering in each group separately.

### 3.2. Discussion

For more than 20 years, the poultry production in Thailand has radically shifted to a more industrial structure, and in 2002 more than 70% of national poultry production was oriented to the export market [13]. Industrial poultry production system in Thailand consists of vertically integrated companies controlling every stage of the production chain, from breeding to marketing processed poultry [21]. Those companies offer contractual agreements to private farms and purchase poultry products from them. The firm holds considerable control over raw material production without ownership of the production units. Contract farmers have an assured market for their poultry, access to the firm’s services, and easier access to credit from banks [22]. Independent farmers mostly sell their larger surpluses to aggregators who drive pickups from village to village, purchasing birds from numerous different farms. A small amount of eggs and live or slaughter birds are sold to “End Users” (neighbors, restaurants or village, market vendors). Due to this production system, 98% of commercial poultry producers (sector 1–3) belongs to sector 2 and 3 in Thailand [3]. Sector 4 still exists as a source of suppliers for local markets.

Our goal was therefore to determine the actual level of vulnerability of this medium and small scale commercial poultry production system (sector 2 and 3) and to determine the most hazardous poultry farming practices related to the risk of avian influenza contamination and spread.

Our study clearly showed that vulnerability among farms in the province of Nakhon Pathom is still very high. It appears that the vulnerabilities related to poor farm management are predominant. Major deficiencies in bio-security measures were found in the areas of farm management, including transport, restocking practice, manure utilization, water management, rodent and insect control, frequency of disinfection, worker routines, hygiene practices and contact between farms. This is undoubtedly the result of a lack of awareness among farmers regarding the potential risks of epidemics. They are relatively complying with current regulations, but not all are entirely convinced that the risk of infections is still real, after a long period without warning since the last outbreak (in 2008).

Easy access to farm through entrances was found in 76% of farms, as the doors are open with no locks or warning sign. This percentage is very high and this is a major issue in vulnerability regarding farm to farm contamination.

75% of farms are located next to a pond or a pond is present at the farm. 19% of farms practice an integrated poultry and fish farming. Droppings and food spilled by chickens fall directly into the water and feed the fish. If birds are housed without proof housing this practice is considered as high risk to disseminate avian influenza virus [6,23,24,25,26]. Furthermore, constant presence of wild birds or ducks attracted by the pond can contribute to the spreading of the disease. Finally, disinfection of poultry houses is not practiced in integrated poultry-fish farms since farmers are afraid of killing the fish. All farmers declare the regular presence of wild birds on the farm. 82% have no proper food storage or keep an open door at poultry food storage.

Although 85% of farmers replied positively to the use of rodent control, 83% of them use cats as the unique means of rodent control. The use of insecticides is very uncommon, and 94% think insects are not an issue. Even if insects themselves do not represent a known risk for the spread of avian influenza, insect infestation is a marker of poor hygiene and poor maintenance of the farm.

The transport of poultry, food, or fomite is an important risk factor for the transmission and dispersion of avian influenza [27,28]. On contractor farms, the company tracks and collects poultry or delivers food directly to the farm, unlike 39% of independent farms who delivers their products to various aggregators. These aggregators move from one farm to another, thus increasing the risk of avian influenza virus entry [29]. This practice represents a significant risk of spreading pathogens between farms if the disinfections of trucks are not applied at the entries and exits of the farm [30]. Similarly, the transport of poultry to the slaughterhouse several times per production cycle and restocking with non-compliance the rule “all in, all out” is also a risk factor [6]. In 93% of visited farms, disinfectant barriers at the farm entrance do not exist. 55% of farms do not practice trucks disinfection. Moreover, at all farms, truck drivers leave vehicles and walk around in their own clothes. These results show a very high vulnerability issue.

Employers of 93% of the farms do not provide or request work clothing, and at 38% of farms the same employees work on several different poultry units. Most farms do not provide food for the workers, and they frequently go out (more than two times a day) to buy a meal. Moreover, 37% of farms permit street food vendors to enter the farms and sell food to workers. Again, these results show a high vulnerability issue regarding virus dissemination. 

Regular cleaning of poultry rearing equipment is practiced on only 26% of farms. Farm disinfection is applied at 59%, and no one in a poultry farm above a fishpond uses disinfectant. Disinfection of poultry houses at the end of the poultry production cycle is widely applied but still not applicable on poultry rearing equipment and egg trays. As animal food, fresh manure is used at 57% of farms, and 50% use dead fowl of farms as animal food.

Farms producing eggs are more vulnerable than farms producing birds and this result is consistent with the type of farms mostly affected during the 2004–2008 outbreaks [2]. Layer farms are more vulnerable than meat farms: factors associated with egg production strongly contributes to the vulnerability, and many farms producing eggs have low results regarding vulnerability factors linked to egg production. 

Although almost all farmers knew about avian influenza, 63% of them do not think it is a dangerous disease and therefore are not concerned. Farmers adhere only to what is requested by the DLD GAP regulations. The long period without warning since the last outbreak of HPAI could also be an element of a relative lessening of farmers’ vigilance. 

Spatial clustering of vulnerability is also an important bio-security issue; infection in a farm may spread to neighboring farms if vulnerability of neighbors is high. Compartmentalization (defined primarily by management and husbandry practices related to biosecurity) is recommended by FAO [6,23] and has been promoted to limit disease spread between farms. Nevertheless, our results show that many vulnerability issues are related to contacts (*i.e.*, easy access to farms, behaviors of workers, low disinfection at entrance) and are clustered. These spatial clusters of vulnerability need to receive special attention from authorities to respect the purpose of compartmentalization.

## 4. Conclusions

The results of our survey in the Nakhon Pathom province has shown that numerous vulnerabilities still exist and could represent a significant risk for large dissemination in the event of HPAI re-emergence. These results strongly suggest that bio-security practices on the majority of farms still need improvement. Particular attention should be conducted when vulnerability is spatially clustered. 

It was also noted that category 3 (bio-security measures and farm management) is the one with the worst average score. In order to improve on-farm bio-security, it is necessary to enhance the capacity of poultry workers and farm owners to recognize the importance of these security measures. This can only be achieved through careful communication with an engagement of farmers and communities. DLD GAP regulations should be sharpened; stricter LFS regulations and GAP formulation with the implication of details is highly recommended for bio-security measures and farm management with a special attention for egg production management and practices.

## References

[1] OIE (2013). Update on Highly Pathogenic Avian Influenza in Animals (Type H5 and H7). http://www.oie.int/en/animal-health-in-the-world/update-on-avian-influenza.

[2] Souris M., Gonzalez J.P., Shanmugasundaram J., Corvest V., Kittayapong P. (2010). Retrospective space-time analysis of H5N1 Avian Influenza emergence in Thailand. Int. J. Health Geogr..

[3] Rushton J., Viscarra R., Bleich E.G., McLeod A. (2007). Impact of Avian Influenza Outbreaks in the Poultry Sectors of Five South East Asian Countries (Cambodia, Indonesia, Lao PDR, Thailand, Vietnam) Outbreak Costs, Responses and Potential Long Term Control. TCP/RAS/3010.

[4] Beaudoin A.L., Kitikoon P., Schreiner P.J., Singer R.S., Sasipreeyajan J., Amonsin A., Gramer M.R., Pakinsee S., Bender J.B. (2012). Risk factors for exposure to influenza a viruses, including subtype H5 viruses, in Thai free-grazing ducks. Transbound. Emerg. Dis..

[5] Costales A. (2002). Thailand Poultry Sector Brief. Food and Agriculture Organization.

[6] (2004). Recommendations on the Prevention, Control and Eradication of Highly Pathogenic Avian Influenza (HPAI) in Asia.

[7] Tiensin T., Chaitaweesub P., Songserm T., Chaisingh A., Hoonsuwan W., Buranathai C., Parakamawongsa T., Premashthira S., Amonsin A., Gilbert M. (2005). Highly pathogenic avian influenza H5N1, Thailand, 2004. Emerg. Infect. Dis..

[8] Gilbert M., Chaitaweesub P., Parakamawongsa T., Premashthira S., Tiensin T., Kalpravidh W., Wagner H., Slingenbergh J. (2006). Free-grazing ducks and highly pathogenic avian influenza, Thailand. Emerg. Infect. Dis..

[9] Paul M., Wongnarkpet S., Gasqui P., Poolkhet C., Thongratsakul S., Ducrot C., Roger F. (2011). Risk factors for highly pathogenic avian influenza (HPAI) H5N1 infection in backyard chicken farms, Thailand. Acta Trop..

[10] Keawcharoen J., Broek J., Bouma A., Tiensin T., Osterhaus A., Heesterbeek H. (2011). Wild birds and increased transmission of highly pathogenic avian influenza (H5N1) among poultry, Thailand. Emerg. Infect. Dis..

[11] Tiensin T., Nielen M., Vernooij H., Songserm T., Kalpravidh W., Chotiprasatintara S., Chaisingh A., Wongkasemjit S., Chanachai K., Thanapongtham W. (2007). Transmission of the highly pathogenic avian influenza virus H5N1 within Flocks during the 2004 epidemic in Thailand. J. Infect. Dis..

[12] (2004). Avian Influenza Situations and Control Measures.

[13] Ranong V.N. (2008). Structural changes in Thailand’s poultry sector: avian influenza and its aftermath. TDRI Quart. Rev..

[14] Wei H., Aengwanich W. (2012). Biosecurity evaluation of Poultry Production Clusters (PPCs) in Thailand. Int. J. Poult. Sci..

[15] Chantong W., Kaneene J.B. (2011). Poultry raising systems and highly pathogenic avian influenza outbreaks in Thailand: the situation, associations, and impacts. Southeast Asia. J. Trop. Med. Public Health.

[16] Bouyer G., Hémon D., Cordier S., Derriernnic F., Stücker I., Stengel B., Clavel J. (1995). Epidemiologie, Principles et Methods Quantitative.

[17] Pfeiffer D., Robinson T., Stevenson M., Stevens K., Rogers D., Clements A. (2008). Spatial Analysis in Epidemiology.

[18] Souris M., Bichaud L. (2011). Statistical methods for bivariate spatial analysis in marked points. Example in spatial epidemiology. Spat. Spatio-Temporal Epidemiol..

[19] Kulldorff M. (1997). A spatial scan statistic. Commun. Statist.—Theor. Method..

[20] SavGIS Geographic Information System. www.savgis.org.

[21] Heft-Neal S., Otte J., Pupphavessa W., Roland-Holst D., Sudsawasd S., Zilberman D. (2008). Supply Chain Auditing for Poultry Production in Thailand. Pro-poor Livestock Policy Initiative Research Report.

[22] Glover D., Kusterer K. (1990). Small Farmers, Big Business: Contract Farming and Rural Development.

[23] FAO/OIE/WHO (2005). Consultation on Avian Influenza and Human Health: Risk Reduction Measures in Producing, Marketing, and Living with Animals in Asia.

[24] Webster R.G., Yakhno M., Hinshaw V.S., Bean W.J., Murti K.G. (1978). Intestinal influenza: Replication and characterization of influenza viruses in ducks. Virology.

[25] Utterback W. Update on Avian Influenza through February 21 1984 in Pennsylvania and Virginia. Proceedings of the 33rd Western Poultry Disease Conference.

[26] Feare C.J. (2006). Fish Farming and the Risk of Spread of Avian Influenza.

[27] Halvorson D.A., Karunakaran D., Newman J.A. (1980). Avian influenza in caged laying chickens. Avian Dis..

[28] Glass S.E., Naqi S.A., Grumbles L.C. (1981). Isolation of avian influenza viruses in Texas. Avian Dis..

[29] Delforge I. (2007). Contract Farming in Thailand: A View from the Farm. Occasional Paper 2, Focus on the Global South, CUSRI.

[30] Thomas M.E., Bouma A., Ekker H.M., Fonken A.J., Stegeman J.A., Nielen M. (2005). Risk factors for the introduction of high pathogenicity Avian Influenza virus into poultry farms during the epidemic in the Netherlands in 2003. Prev. Vet. Med..

